# AZD5153 enhances the chemo-sensitivity of gemcitabine on pancreatic cancer cells in vitro and in vivo

**DOI:** 10.1186/s12935-025-03952-2

**Published:** 2025-08-26

**Authors:** Haixin Zhu, Minmin Shen, Yiqian Zhu, Ruoqi Wang, Rong Dong, Yuyu Huang, Lulin Zhu, Ying Li, Youyou Yan, Jiang Lou, Bo Zhang, Nengming Lin, Biqin Tan

**Affiliations:** 1 College of Pharmaceutical Sciences, Hangzhou First People’s Hospital, Zhejiang Chinese Medical University, Hangzhou, Zhejiang China; 2 Present Address: Department of Pharmacy, Key Laboratory of Clinical Cancer Pharmacology and Toxicology Research of Zhejiang Province, Affiliated Hangzhou First People’s Hospital, School of Medicine, Westlake University, Hangzhou, 310006 China; 3 Department of Drug Clinical Trial Institution, Huzhou Central Hospital, Huzhou, 313000 Zhejiang China; 4 The Affiliated Hospital of Hangzhou Normal University, Hangzhou, 310006 Zhejiang China; 5 Department of Pharmacy, Tongde Hospital of Zhejiang Province, Hangzhou, PR China; 6 Westlake Laboratory of Life Sciences and Biomedicine of Zhejiang Province, Hangzhou, 310024 China; 7 Cancer Center, Zhejiang University, Hangzhou, 310058 China

**Keywords:** AZD5153, Gemcitabine, Pancreatic cancer, ERK/mTOR signaling, MUC2

## Abstract

**Background:**

Pancreatic cancer is a malignant disease with a poor prognosis. Gemcitabine (GEM), the first-line treatment drug, shows limited efficacy because of the notorious drug resistance of pancreatic cancer. Therefore, the development of sensitive drugs for pancreatic cancer is essential. AZD5153 is a novel bivalent BET bromodomain inhibitor with multiple anti-tumor effects on malignancy. Here, we aimed to investigate the effect of AZD5153 on the GEM sensitivity in human pancreatic cancer cells.

**Methods:**

Sulforhodamine B (SRB), clone formation assays were designed to characterize the cell viability and clone formation after treatment with AZD5153 and/or GEM. DAPI staining, flow cytometry and western blotting were used to identify the cell apoptosis. RNA-seq analysis, western blotting and qPCR were also conducted to confirm the signaling pathway involved in it. Nude mice bearing PANC-1 pancreatic cancer xenograft model was conducted to confirm the combination effect of GEM and AZD5153 in vivo.

**Results:**

As a result, AZD5153 presented a strong anti-proliferation activity and exerted synergistic effects when combined with GEM in BXPC3 and PANC-1 cell lines.Meanwhile, the combination treatment also inhibited colony formation in these two cell lines. Additionally, AZD5153 combined with GEM induced cell apoptosis. Further investigations revealed that the combination of AZD5153 and GEM decreased the phosphorylation of ERK/mTOR signaling proteins, the specific chemical activators PDBu (activator of ERK) reversed the expression of c-PARP. Besides, the expression of MUC2 was remarkable decreased after combination treatment.

**Conclusion:**

In conclusion, these results suggested that AZD5153 might be an excellent GEM sensitizer in pancreatic cancer.

**Supplementary Information:**

The online version contains supplementary material available at 10.1186/s12935-025-03952-2.

## Introduction

Pancreatic cancer is one of the most common malignancies worldwide and has a poor prognosis [1–3]. The mortality rate of pancreatic cancer in China ranks sixth among all cancers in males (eighth in females), according to Cancer Statistics 2022 from the National Cancer Center in China. Traditional treatments, including surgery, radiotherapy, and chemotherapy, improve the outcomes of patients [4, 5]. Late diagnosis makes many patients unsuitable for surgery. Chemotherapy is the most important treatment for patients who are not able to undergo resection. However, gemcitabine (GEM)-based regimens, which serves as the cornerstone of treatment, shows limited efficacy because of the notorious drug resistance of pancreatic cancerand contributes to the poor prognosis [6–8]. Therefore, novel therapeutic strategies must be identified to improve the effectiveness of GEM treatment in patients with pancreatic cancer.

AZD5153 is a novel bivalent BET bromodomain inhibitor with potent anti-tumor effects on malignant cancer cells [9, 10]. AZD5153 inhibited proliferation and induced apoptosis in HCT116 and LoVo colorectal cancer cells, and further experiments indicated that AZD5153 sensitized cells to the antitumor effect of the PARP inhibitor BMN673 in vitro and in vivo [11]. Garrett W. Rhyasen et al. showed that AZD5153 inhibited BRD4 expression to subsequently affect the transcriptional programs of MYC and E2F, indicating an anticancer effect on hematological malignancies [9]. In PC3 prostate cancer cells and primary prostate cancer cells, AZD5153 induces apoptosis and down-regulates the expression of BRD4 target genes. Meanwhile, AKT may be the primary factor influencing AZD5153 resistance in PC3 cells [12]. Other studies have indicated that the effect of AZD5153 is related to depolarizing M2 macrophages and DNA damage [13–16]. However, its therapeutic potential and effect on GEM sensitivity have not been fully studied in pancreatic cancer. Given the pressing need for more effective treatments in pancreatic cancer and the multifaceted anti-tumor properties of AZD5153, we designed this project to investigate the anticancer effect of AZD5153 on pancreatic cancer.

In the present study, we aimed to determine the efficacy of AZD5153 on sensitive GEM in pancreatic cancer cell lines in vitro and in vivo.,and explore the possible regulatory machanism involved in it. The study suggest an effective strategy for enhancing the effect of GEM and provide insights into the development of AZD5153.

## Materials and methods

### Chemicals and cell culture

AZD5153 was purchased from Selleck, USA. The chemical was dissolved in DMSO (Sigma–Aldrich, USA) and stored at −20 °C. The Fucoidan(FU, HY-132179), lipopolysaccharides (LPS, HY-D1056) and Cholic acid (DCA, HY-N0324) were purchased from MCE. The human pancreatic cancer cell lines BXPC3 and PANC-1 were obtained from Stem Cell Bank, Chinese Academy of Sciences, and routinely maintained in RPMI-1640 medium (HyClone, USA) supplemented with 10% FBS plus 1% penicillin-streptomycin. All cells were incubated at 37 °C in a 5% CO2 atmosphere.

### Cell proliferation assay

Cell viability was measured using a sulforhodamine B (SRB, Sigma Aldrich) assay. Briefly, 3–5 × 10^3^ cells were cultured in 96-well plates. After 24 h, the cells were treated with the indicated concentrations of AZD5153 and/or GEM, and cell viability was measured at the indicated times. Absorbance was measured at 450 nm using a Multiskan Spectrum spectrophotometer (Thermo Scientific, Rockford, IL, USA).

### Colony formation assay

BXPC3, PANC-1 cells were seeded in 6-well plates at a density of 600 cells/well. After 24 h incubation, the cells were then incubated with AZD5153 and/or GEM. Following another 48 h treatment, the supernatant was replaced regularly for 2 weeks. the cells were finally fixed with crystal violet solution (0.1%), colonies were photographed.

### DAPI staining

BXPC3 and PANC-1 cells were seeded in 6-well plates, exposed to AZD5153 and/or GEM. The cells were harvested, fixed for 30 min (pre-cooled 4% paraformaldehyde), and stained with DAPI for 20 min. 1×PBS were used for washing three times, the cells were photographed using a fluorescence microscope (Nikon, Japan) [17, 18].

### Apoptosis assay

BXPC3 and PANC-1 cells (5 × 10^5^/ml) were seeded into 6-well plates, treated with AZD5153 and/or GEM for 24 h and harvested. PBS was used to wash. Then Annexin V-FITC (Invitrogen, Carlsbad, CA) and propidium iodide (PI) were added according to the protocol and analyzed by flow cytometry (Becton Dickinson, Franklin, NJ, USA).

### Western blotting analysis

After treatment with AZD5153 and/or GEM, total protein was extracted. A total protein sample (40 µg) was conducted to electrophoresis. The proteins were transferred to a PVDF membrane (Bio-Rad, USA) with indicated time, stained with 5% nonfat milk for 1 h at room temperature. Primary antibodies were stained overnight at 4 °C. The primary antibodies were as follows: p-AKT (Ser473, CST, 12694, 1:1000), AKT (CST, 9272, 1:1000), p-mTOR (Ser2448, CST, 5536, 1:1000), mTOR (CST, 5536, 1:1000), p-p70S6K (Thr389, CST, 9234, 1:1000), p70S6K (CST, 2708, 1:1000), pS6 (Ser240/244, CST, 5364, 1:1000), S6 (CST, 2217, 1:1000), p-ERK1/ERK2 (Abcam, ab50011, 1:5000), ERK1/ERK2 (Abcam, ab17942, 1:5000), Caspase-3 (CST, 9662, 1:1000), Bcl-2 (Abcam, ad324, 1:5000), PARP (CST, 9542p, 1:1000), Cleaved PARP (CST, 5625 s, 1/1000), Cleaved Caspase-3 (CST, 9661 s, 1/1000), BRD4 (CST, 13440, 1:1000), MUC2 (ABclonal, A14659, 1:1000), JAK2 (Abcam, ab32101, 1/1000), P-JAK2 (CST, 3291 T, 1/1000), STAT3 (CST, 9139 s, 1/1000), p-STAT3 (CST, 9145 s, 1/1000), β-Tubulin (9F3, CST, 2128, 1:1000), C-MYC (CST, 18583 S, 1/1000), GAPDH (Santa Cruz, sc-32233, 1:1000), and β-Actin (Santa Cruz, sc-47778, 1:1000). Then, the secondary antibodies were used to stain at room temperature for 1 h. The protein were detected by ECL incubating. The blots we performed were cut prior to hybridisation with antibodies.

### Animal treatment and drug administration

4-week-old female nude mice were purchased from the Shanghai JieSiJie Laboratory Animal Co., Ltd.All animal experiments were conducted according to the Institutional Animal Care and Use Committee (IACUC). BXPC3 cells (4 × 10^6^) were re-suspended, subsequently injected into 4-week-old female nude mice. When the tumors reached ~ 90 mm^3^, the mice were randomized into different groups: vehicle, AZD5153 (5 mg/kg), gemcitabine (20 mg/kg) and combination groups. The mice received the following drug treatments every two days for 15 days. At the end of the 15 days, the mice were euthanized using a rate of 20–30% CO_2_ chamber volume per minute. Tumor volume (TV) was determined from (length × width^2^)/^2^.

### RNA-Sequencing analysis

BXPC3 cells were treated with or without GEM, AZD5153, and total RNA was extracted with Trizol reagent following the manufacturer’s procedure. After purification and quantification, the cleaved RNA was reverse transcribed to create the cDNA by SuperScript™ II Reverse Transcriptase (Invitrogen, 1896649), which were next used to synthesise U-labeled second-stranded DNAs and used for the construction of sequencing libraries. The average insert size for the final cDNA library were 300 ± 50 bp. At last, we performed the 2 × 150 bp paired-end sequencing (PE150) on an Illumina Novaseq™ 6000 (LC Bio Technology Co., Ltd., Hangzhou, China) following the vendor’s recommended protocol.

Genes differential expression analysis was performed by edgeR between two samples. The genes with the parameter of p value < 0.05 and |fold change|≥2 were considered differentially expressed genes (DEG).

To identify the most significantly enriched pathways among the DEGs, we performed Gene Ontology (GO) and Kyoto Encyclopedia of Genes and Genomes (KEGG) pathway analyses using clusterProfiler (3.10.1) and org.Hs.eg.db (3.7.0).

Gene set enrichment analysis (GSEA) was performed to identify functionally relevant gene clusters using ClusterProfiler. Gene sets were obtained from MSigDB (https://www.gsea-msigdb.org/gsea/msigdb/human/collections.jsp#H),, including Hallmark, JAK-STAT3 signaling (HALLMARK_IL6_JAK_STAT3_SIGNALING).

### Quantitative real-time (qPCR)

Total RNA from cells was extracted using TRIzol reagent (Invitrogen). RNA was reverse transcribed into cDNA. qPCR was performed on a 7500 Fast System using SYBR-Green (Qiagen, Hilden, Germany) and a volume of water to generate a volume of 20 µl. Assays were performed on three independent experiments. The primers used was as follows:

MUC2 Forward: TGTGTTTCAGGCTCCATCAC.

MUC2 Reverse: TGCAGCCATTGTAGGAAATC.

FGFBP1 Forward: AACTGCAGATGGGCTGCTAC.

FGFBP1 Reverse: TCTCCTTCCTGGGCTTTGTG.

GSDMC Forward: GAGGGGACAACCTGTACGTG.

GSDMC Reverse: TCTGAAGAGTCAGCGCCTTC.

### RNA interference

The cells were seeded in 6-well plates, cultured for 12 h. Transfection of siRNA was performed using Lipofectamine 2000. After 6 h to eliminate the toxic effects of transfection, the medium was replaced with complete medium after 8 h. The cells were cultured for 24 h, and then treated with indicated drugs. The cells were harvested for protein extraction.

### Statistical analysis

The collected data were calculated as the means ± SD/SE. at least three independent experiments. Differences between groups were performed with Student’s t-test and *p < 0.05 was defined as significant difference. Graphs were prepared using GraphPad software and SigmaPlot 14. The IC_50_ values were determined using GraphPad Prism software by fitting a dose-response curve (nonlinear regression).

## Results

### AZD5153 pretreatment enhanced the effect of GEM on pancreatic cancer cells

**Fig. 1:**
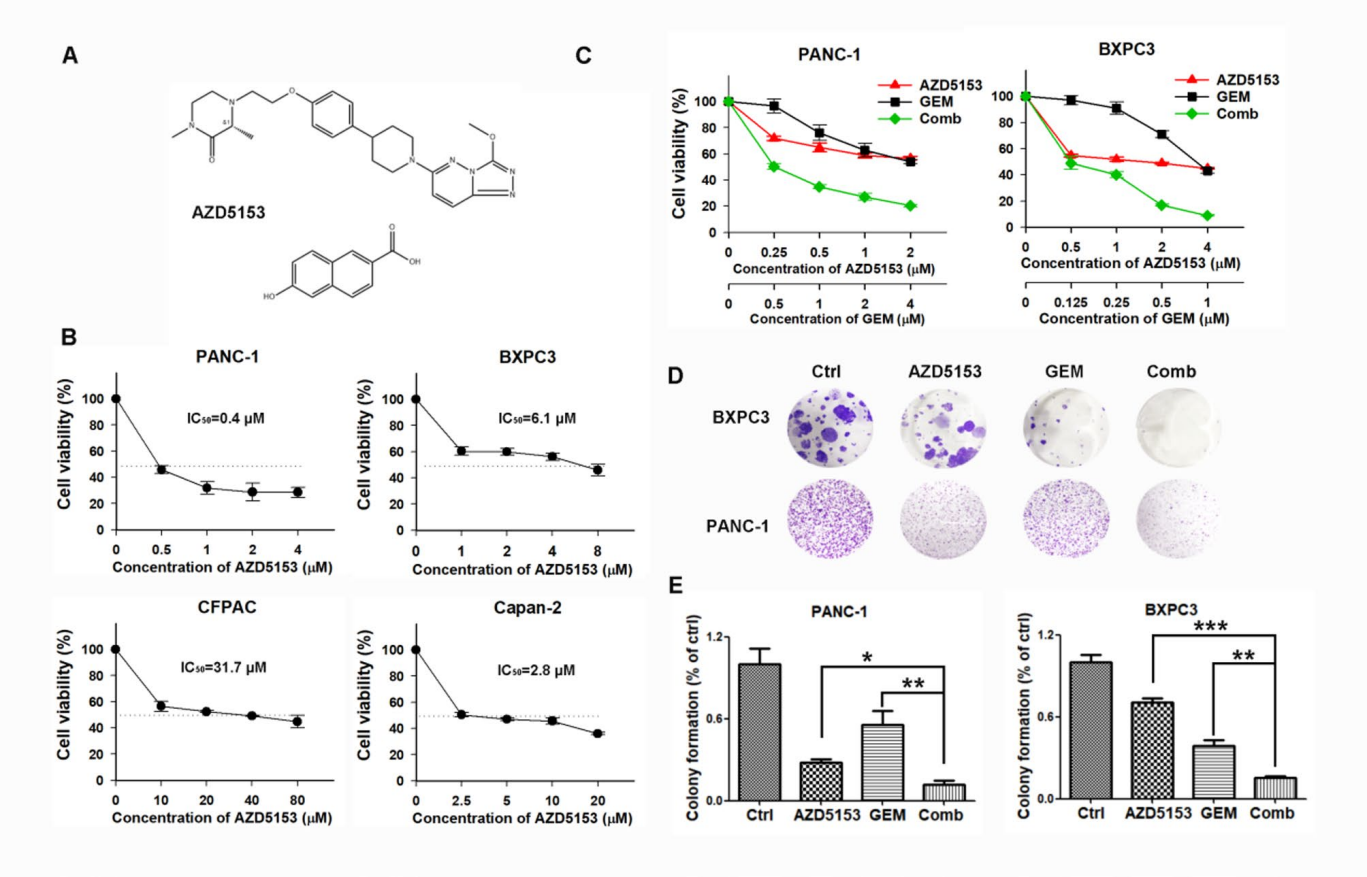
AZD5153, GEM, and their combination inhibited cell proliferation in vitro. (**A**) The chemical structure of AZD5153. (**B**) A SRB assay was performed to detect the inhibitory effect of AZD5153 on the proliferation of the pancreatic cancer cell lines PANC-1, BXPC3, CFPAC, and Capan-2 after treatment for 48 h. (**C**) BXPC3 and PANC-1 cells were treated with PBS, AZD5153, GEM, or their combination for 48 h, and then the absorbance was measured. (**D**) Effects on the colony formation of BXPC3 and PANC-1 cells. BXPC3 or PANC-1 cells were seeded in 60-mm dishes and treated with AZD5153 (2 µM for BXPC3, 0.5 µM for PANC-1), GEM (0.5 µM for BXPC3, 1 µM for PANC-1) or combine for 14 days. The colonies were stained and photographed. (**E**) Histograms show the number of colonies. The results are presented as the means ± SD of 3 independent experiments. Significance was determined using Student’s t-test (*p < 0.05, **p < 0.01, ***p < 0.001 compared with combination-treated cells)

First, we investigated the effect of AZD5153 (the chemical structure is shown in Fig. 1A) on the proliferation of pancreatic cancer cell lines using the sulforhodamine B (SRB) assay. Cells were cultured in 96-well plates for 24 h and then treated with the indicated concentrations of AZD5153. Cell viability was detected at 48 h. As shown in Fig. 1B, AZD5153 reduced the viability of pancreatic cancer cells in a dose-dependent manner. The IC_50_ values for AZD5153 at 48 h of treatment were 6.1 µmol/L, 2.8 µmol/L, 31.7 µmol/L, and 0.4 µmol/L in BXPC3, Capan-2, CFPAC and PANC-1 cells, respectively. Furthermore, the sensitivities of BXPC3 and PANC-1 cell lines to AZD5153, GEM, or their combination were determined. Viability curves for cells treated with AZD5153, GEM, and AZD5153 combined with GEM are shown in Fig. 1C. CI values were calculated using Calcusyn at fixed-ratio concentrations of AZD5153 and GEM to assess the activity of the combination treatment. AZD5153 plus GEM exerted a synergistic effect on two human cancer cell lines, with mean CI values below 0.5 (STable 1). The same results were obtained in CFPAC and Capan2 cells (Fig. S1). Thus, the combination was much more effective than either single agent at inhibiting the proliferation of human cancer cells. Similarly, colony formation assays showed that the combination treatment inhibited colony formation by BXPC3 and PANC-1 cells more obviously than either agent alone (*p* < 0.05) (Fig. 1D and E).

### Treatment with AZD5153 in combination with GEM induced apoptosis in pancreatic cancer cells

**Fig. 2:**
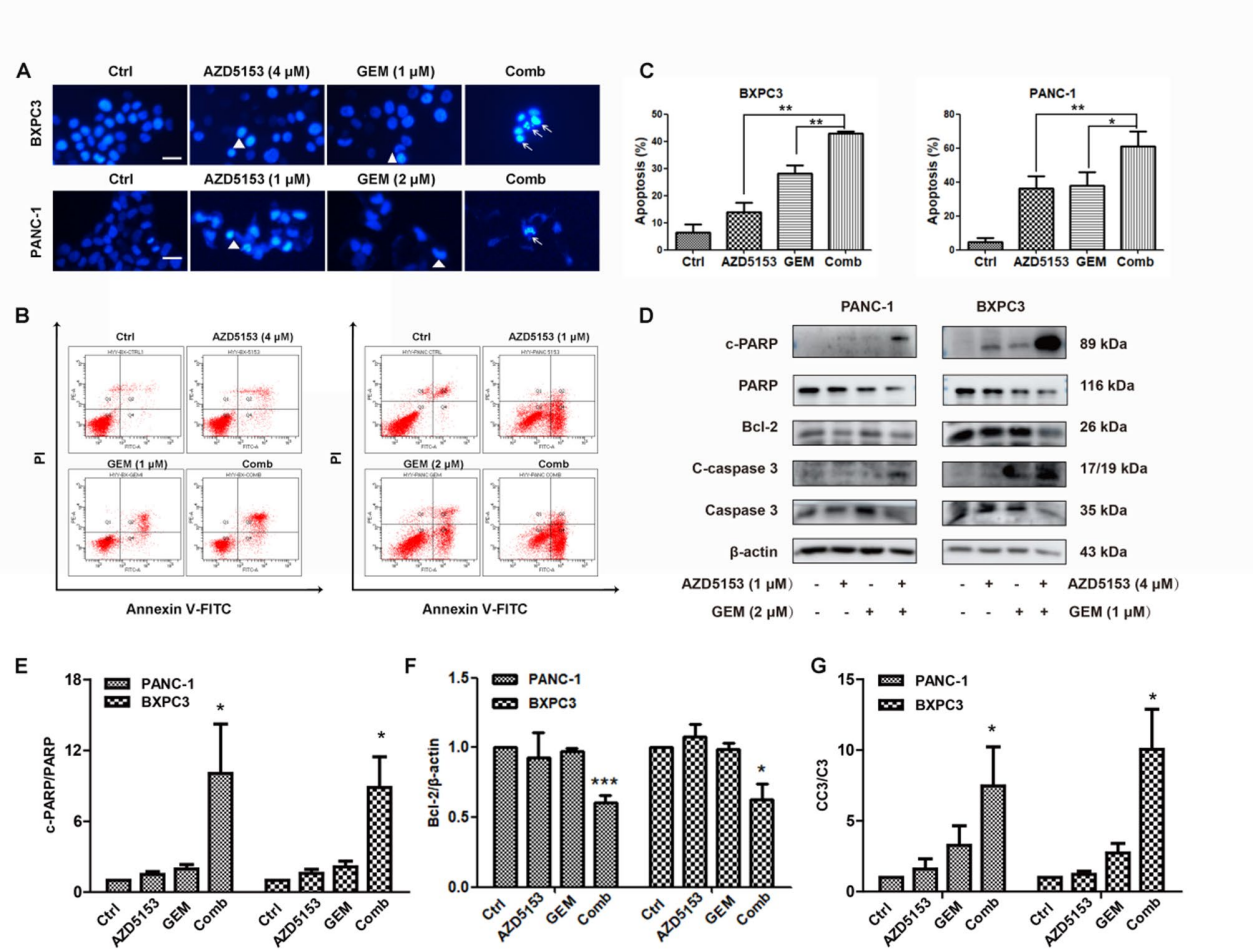
AZD5153 and GEM synergistically induce apoptosis in pancreatic cancer cells. (**A**) BXPC3 and PANC-1 cells were exposed to AZD5153, GEM, or their combination at the indicated concentrations for 24 h. After exposure, the cells were stained with DAPI, and the apoptotic nuclear changes were examined under a fluorescence microscope. Triangles indicate chromatin condensation, white arrows indicate nuclear collapse. (**B**) Human BXPC3 and PANC-1 cells were exposed to AZD5153, GEM, or their combination for 48 h. The cells were then stained with Annexin V-FITC and PI and detected using flow cytometry. (**C**) The percentages of apoptotic cells in each group were quantified. (**D**) Western blotting was performed to detect the expression of the apoptosis-related proteins Bcl-2, PARP, c-PARP, c-Caspase 3 and Caspase 3. β-actin was used as a loading control. (**E-G**) The ratios of c-PARP/PARP, Bcl-2/β-actin, CC3/C3 were quantified by densitometry based on immunoblot images. The results are presented as the means ± SD of 3 independent experiments. Significance was determined using Student’s t-test (*p < 0.05, **p < 0.01, ***p < 0.001 compared with the combination group in C; *p < 0.05, ***p < 0.001 compared with the Ctrl group in E-G)

Apoptosis assays were conducted using Hoechst staining, flow cytometry and western blotting to identify the role of AZD5153 and GEM in inducing cell apoptosis. Cell morphological changes were examined under a microscope following DAPI staining. In control cells, the nuclei are uniform and have smooth nuclear membranes, the cells treated with AZD5153 and/or GEM exhibited early apoptosis’s morphological features, such as apoptotic bodies, nuclear chromatin condensation and bright staining (Fig. 2A). Annexin V-PI double staining was utilized to further demonstrate cell apoptosis after AZD5153 and/or GEM treatment. AZD5153 (13.9 ± 3.64%) and GEM (28.13 ± 2.92%) increased apoptosis at 48 h, and the combination treatment (42.93 ± 0.76%) significantly increased the apoptosis ratio compared with the control treatment (6.4 ± 3.12%) in BXPC3 cells. Similar results were obtained using PANC-1 cells. This result indicated that AZD5153 enhanced GEM-induced apoptosis. We examined the expression of apoptosis-related proteins to validate our results. The expression of PARP, Bcl-2 and Caspase 3 were decreased, while the c-PARP and c-Caspase 3 were increased after treatment with the combination of AZD5153 and GEM (Fig. 2D-G). Taken together, these results clearly suggest that AZD5153 and GEM induced apoptosis in pancreatic cancer cells.

### AZD5153, GEM and their combination inhibited tumor growth in vivo

**Fig. 3:**
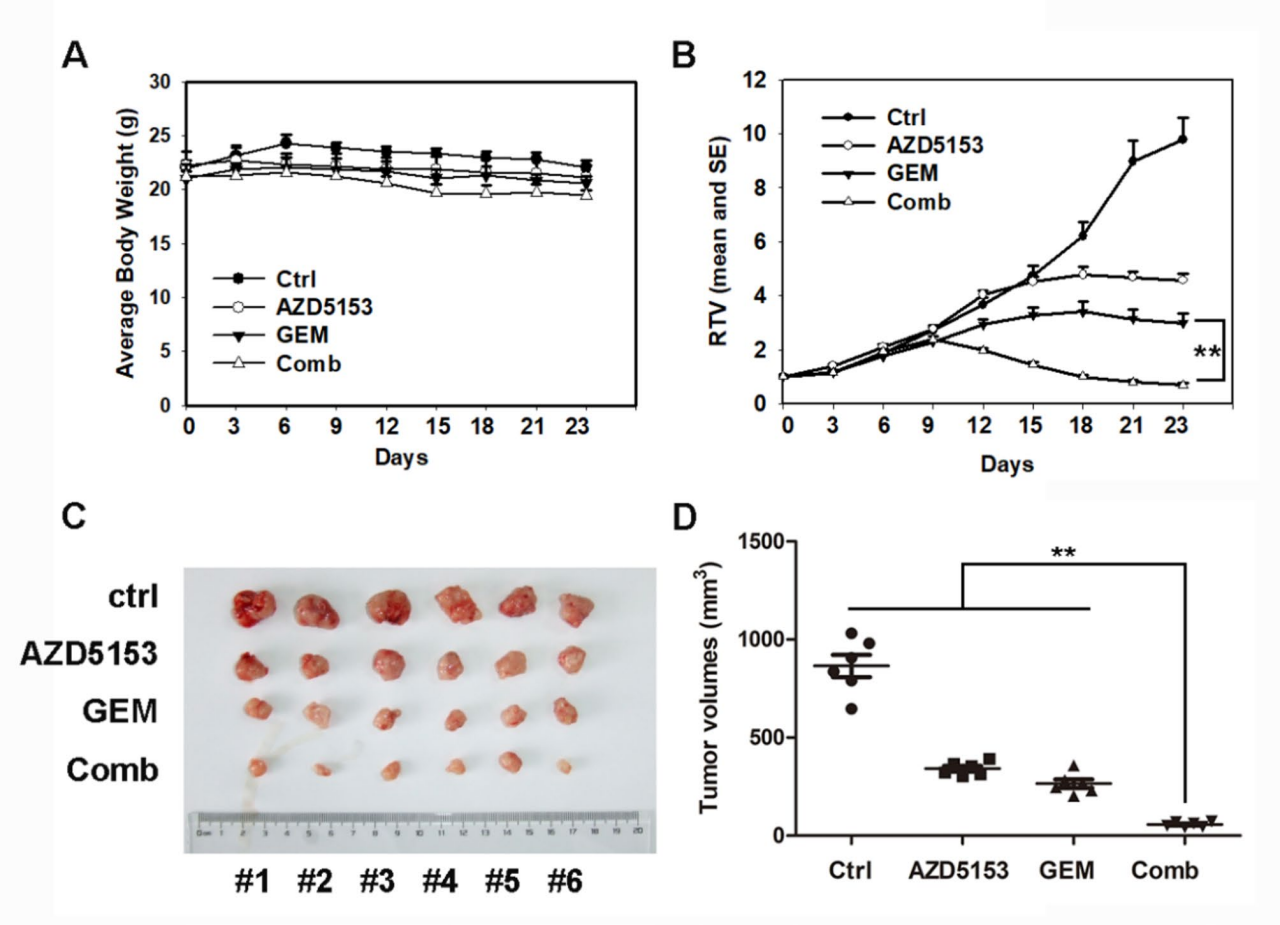
AZD5153, GEM, and their combination suppressed tumor growth in vivo. (**A**) Mice transplanted with PANC-1 xenografts were randomly divided into 4 groups (*n* = 7 mice/group) and treated with the following compounds for 15 days: vehicle, AZD5153 (5 mg/kg), GEM (20 mg/kg), and their combination. The RTV is presented as the means ± SD. (*n* = 7, compared with Ctrl/Comb, one-way ANOVA with Tukey’s multiple comparisons test). The xenograft tumor size was monitored every three days. The body weights are shown in panel A. Tumor volumes were determined using the formula (L×W2)/2. The relative tumor volume (RTV) was calculated using the following formula: RTV=(tumor volume on measured day)/(tumor volume on Day 0) (**B**). (**C-D**) Representative photograph of implanted tumors in each mouse group at the termination time. The tumor volumes were indicated at the termination time (**D**). Data are presented as the means ± SD. **p < 0.01

Based on the in vitro synergistic effect of AZD5153 and GEM, we analyzed the in vivo anti-tumor activity of the combination therapy in nude mice bearing PANC-1 pancreatic cancer xenografts. The mice inoculated with PANC-1 cells were randomly divided into four groups (7 mice per group). After 15 days of treatment with the vehicle, AZD5153, GEM, or combination, the tumors were collected. As indicated in Fig. 3A, compared with the initial body weights, combination-treated mice showed no significant body weight loss on Day 15. AZD5153 and GEM alone, as well as their combination, significantly decreased the xenograft tumor volume (*p* < 0.05) (Fig. 3A-B). The average tumor volume in the combination group was significantly lower than those in the other groups at termination. (Fig. 3C-D). Additionally, in the end of the experiment, we detected the expression of apoptosis-related proteins in tumor tissues. AZD5153 or GEM alone and their combination reduced the expression of PARP and Caspase 3, while the expression of c-PARP, c-Caspase 3 were increased compared with the control (Figure S2). Thus, the combination of AZD5153 and GEM produced much more potent tumor growth inhibitory effects without increasing toxicity to the animals.

### AZD5153 combined with GEM suppressed erk/mtor phosphorylation

**Fig. 4:**
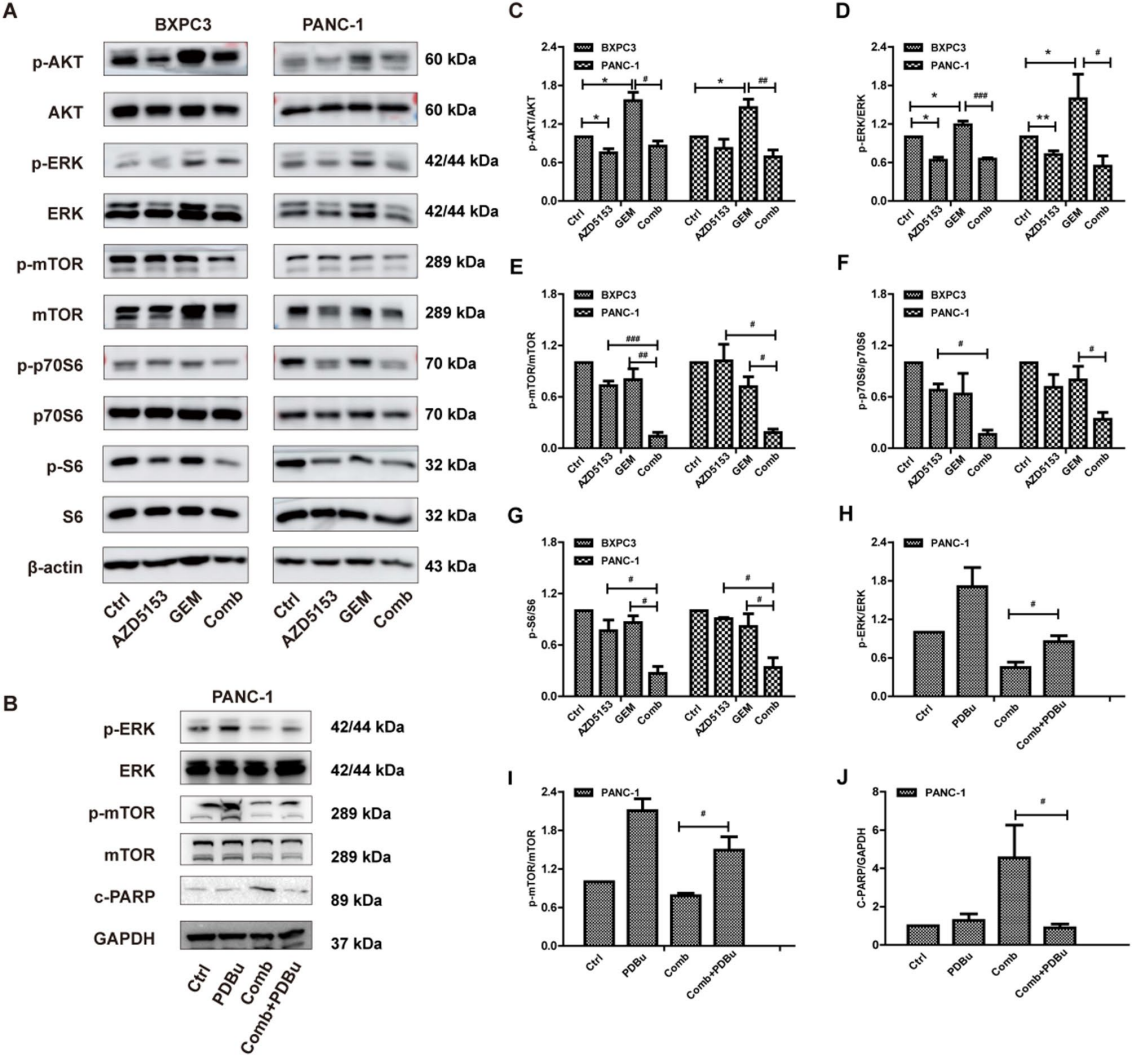
AZD5153 combined with GEM induced apoptosis through a mechanism mediated by the ERK/mTOR signaling pathway. (**A**) Cells were treated with AZD5153, GEM, or their combination for 24 h. Western blotting analysis was performed to detect the levels of mTOR, p-mTOR, p70s6, p-p70s6, AKT, p-AKT (S473), S6, p-S6, ERK, p-ERK, and β-actin, the loading control. (B) Cells were pretreated with PDBu and then treated with AZD5153 and GEM for 24 h. Western blotting analysis was performed to detect the expression of p-ERK, ERK, p-mTOR, mTOR (**B**), c-PARP (**C**), and GAPDH was used as a loading control. (**C-J**) The ratios of p-AKT/AKT, p-ERK/ERK, p-mTOR/mTOR, p-p70S6/p70S6, p-S6/S6, c-PARP/β-actin were quantified by densitometry based on immunoblot images. Results shown are the means SD of three independent experiments. Significance was determined by One-way ANOVA analysis (*p < 0.05, **p < 0.01, ***p < 0.001 vs. Ctrl group; ^#^*p* < 0.05, ^##^*p* < 0.01, ^##^*p* < 0.001 vs. Comb group)

AKT/mTOR, ERK/mTOR is associated with GEM resistance in PDAC. To determine the underlying mechanism of the AZD5153 and GEM combination therapy, so we analyzed the level of the associated proteins’ expression in BXPC3 and PANC-1 cells. As shown in Fig. 4A, BXPC3 cells treated with 4 µM AZD5153 or 1 µM GEM showed a slight decrease in p-ERK, p-AKT, p-mTOR, p-p70S6 and p-S6 levels. Moreover, the combination of AZD5153 and GEM resulted in a stronger reduction in the phosphorylation of these proteins compared with AZD5153 or GEM alone. Similar results were also obtained using PANC-1 cells (1 µM AZD5153 and 2 µM GEM, Fig. 4A and C-G). To further confirm the role of AKT and ERK in the cell apoptosis, the specific chemical activators SC79 (activator of AKT) and PDBu (activator of ERK) were used. The addition of PDBu decreased the expression of c-PARP, however, the SC79 could not decreased the c-PARP expression (Fig. 4B, H-J, Fig. S3).

Besides, AZD5153 is a bivalent BRD4 inhibitor targeting two bromodomains of BRD4, in our study the expression of BRD4 was unchanged by AZD5153 in BXPC3 and PANC-1 cell lines, but its important transcriptional regulator C-MYC is downregulated both in AZD5153 and Combination group (Fig. S4). Collectively, these data suggested that the apoptotic effect of AZD5153 in combination with GEM may be mediated by the inhibition of ERK/mTOR signaling in pancreatic cancer cells.

### MUC2 is down-regulated after AZD5153 and GEM treatment

**Fig. 5:**
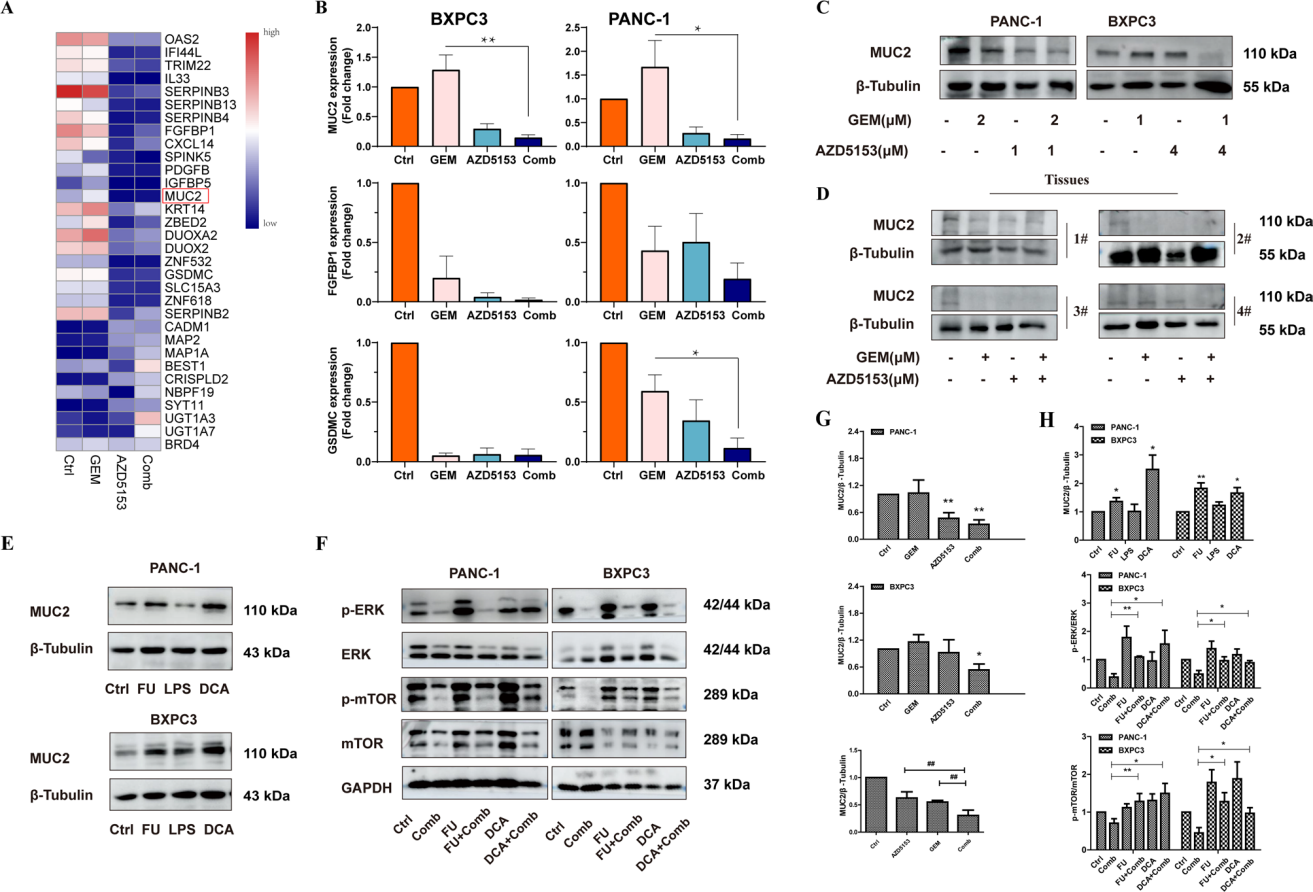
AZD5153 combined with GEM decreased the expression of MUC2. (**A**) Differential gene expression results from RNA-sequencing data. (**B**) qPCR was performed to detect the expression of MUC2, FGFBP1 and GSDMC. (**C** and** D**) Western blotting analysis was performed to detect the expression of MUC2 in cells and tissues, and β-tubulin was used as a loading control. (**E**-**F**) Western blotting analysis was performed to detect the expression of MUC2, p-ERK, ERK, p-mTOR, mTOR, and GAPDH was used as a loading control. (**G**-**H**) The ratios of MUC2/β-actin were quantified by densitometry based on immunoblot images. The results are presented as the means ± SD of 3 independent experiments. Significance was determined by One-way ANOVA analysis (*p < 0.05, **p < 0.01 vs. Ctrl group; ^##^*p* < 0.01 vs. Comb group)

To further find out the key proteins that play critical roles in the synergistic effect of AZD5153 combined with GEM, mRNA sequencing was performed to identify the genes that differentially expressed in BXPC3 cells treated with AZD5153 or combined with GEM. A total of 2933 mRNAs (1365 and 1568 mRNAs were up-regulated and down-regulated respectively) were shown to be significantly changed between combination and control group (Figure S5A). Five potential genes were selected for further study, and the expression of genes were down-regulated after combination treatment (Fig. 5A, Figure S5B). Only MUC2, FGFBP1 and GSDMC were decreased after combination treatment in qPCR analysis (Fig. 5B). Notably, MUC2 was the most significantly down-regulated gene among these detected genes in WB (Fig. 5C-D, G). To further validate the role of MUC2 in this process, we selected three MUC2-upregulating drugs (FU, DCA, and LPS) and treated BXPC3 and PANC-1 cells with them. As shown in Fig. 5E, all three drugs upregulated MUC2 protein expression to varying degrees. When AZD5153 and GEM were administered simultaneously, it was observed that drug-induced overexpression of MUC2 could reverse the expression of p-ERK and p-mTOR proteins caused by the combination treatment (Fig. 5F, H). Knockdown of MUC2 using siRNA and subsequent detection of pathway protein changes yielded consistent results (Figure S8). These results suggest that MUC2 may be a potential target genes in AZD5153 and GEM group. However, much more research should be made to identify it.

## Discussion

In our present study, we indicated that AZD5153 enhanced the effect of GEM on BXPC3 and PANC-1 cells in vitro and in vivo. These effects of AZD5153 were attributable to cell apoptosis, which resulted from the inhibition of the ERK/mTOR signaling pathway. Besides, the expression of MUC2 is decreased obviously after combination treatment. Also, AZD5153 and GEM together inhibited the JAK2-STAT3 signaling pathway (Figure S5). Based on these results, AZD5153 functions as a potent GEM sensitizer in the treatment of pancreatic cancer.

Gemcitabine, used alone or in combination with other agents, is the first-line chemotherapy strategy for the treatment of pancreatic cancer [19, 20]. However, many patients do not benefit from gemcitabine and develop chemoresistance to it [5, 21]. Many efforts have been made to improve the efficacy of gemcitabine [22–27]. Oblongifolin C suppresses tumor growth by downregulating Src and enhances the chemosensitivity of GEM-resistant PC [28]. The administration of MA242 (dual inhibitor of MDM2 and NFAT1) in combination with gemcitabine inhibits pancreatic tumor growth and metastasis, providing a promising strategy for the treatment of pancreatic cancer [29]. However, an effective measure is still unavailable for clinical application. Researchers should continue to explore the appropriate targets or drugs to improve the clinical effects.

The targeted therapy plays an important role in Hematologic Tumors. In recent years, the targeted genes in hematological tumors also have an important function in solid tumors [30–35]. We selected three important target genes (BTK, SYK and BRD4), and tested the anti-tumor effects of their inhibitors on pancreatic cancer cell lines. R406 (BRK inhibitor) and ACP196 (BTK inhibitor) showed little anti-tumor effect alone or together with GEM (Figure S1). AZD5153 is a novel BRD4 inhibitor. AZD5153 mechanically and simultaneously connects to two BRD4 bromodomains, which is a different mechanism from the previous BRD4 inhibitors (such as JQ1 [34]), and exerts potent anti-tumor effects on a variety of cancers (such as hematological malignancies, prostate cancer, and colorectal cancer). A first-in-human, phase I study investigated AZD5153 alone or with olaparib in patients, with relapsed/refractory solid tumors or lymphoma (NCT03205176). This clinical trial has been completed and showed promising results [36]. AZD5153 is also being studied in two other clinical trials (NCT03527147, NCT03013998), which indicated that AZD5153 was tolerable as monotherapy and in combination at the RP2Ds; common toxicities were fatigue, hematologic AEs, and gastrointestinal AEs, therefore AZD5153 has important clinical value and application prospects. In our study, we explored the effects of AZD5153 and gemcitabine in pancreatic cancer cells. The results showed that AZD5153 increased the gemcitabine sensitivity of pancreatic cancer cells in vitro and in vivo.

To further explore the underlying molecular mechanisms responsible for the combination effect of AZD5153 and gemcitabine, we first analysed the drug resistance machanisms of gemcitabine. p-AKT potentially serves as a prognostic and predictive biomarker of PDAC [37]. The AKT inhibitor MK-2206 sensitizes human pancreatic cancer cells to gemcitabine [38, 39]. Epigallocatechin-3-gallate (EGCG) synergizes with gemcitabine to suppress pancreatic cancer cell growth, migration, and invasion by modulating the expression of EMT markers and inhibiting the Akt pathway [40]. In addition, pharmacological inhibition or genetic depletion of AKT induces BRD4 downregulation, sensitizing PC-3 cells to AZD5153-induced cytotoxicity [12]. GINS2 could stimulating the ERK/MAPK signaling to promote the pancreatic cancer development. Researchers have not yet determined whether AZD5153 alters the levels of p-AKT or p-ERK and downregulated genes in pancreatic cancer cells. In our study, AZD5153 combined with gemcitabine decreased the levels of p-AKT, p-ERK and p-mTOR and downregulated genes in BXPC3 and PANC-1 cells. However, the activor of ERK (PDBu) could decreased the expresison of c-PARP induced by GEM and AZD5153, the SC79 (activator of AKT) could not. These data indicated that the effects of AZD5153 combined with gemcitabine were likely at least partially attributable to the inhibition of the ERK/mTOR signaling pathway.

AZD5153 did not decrease BRD4 expression in BXPC3 and PANC-1 cells in our research. Further RNA-seq analysis found that the expression of MUC2 was down-regulated after combination treatment. MUC2 is a secretory mucin that is expressed in small intestine, colon and other organs. It is abnormally expressed in malignant tumors. Some studies demonstrated that MUC2 acted as a tumor suppressive genes, MUC2 mRNA levels was significantly decreased in the colon cancer compared with the control group (decreased by 30%, respectively) [41, 42]. However, Cardillo MR et al. reported that the immunohistochemical expression level of MUC2 is significantly elevated during the transition of primary bladder cancer to a more aggressive phenotype. High MUC2 staining was observed in patients with moderately differentiated tumorsIn addition, MUC2 methylation also plays a significant role in this process [43–46]. In our study, qPCR and western blotting indicated the down-regulated MUC2 at the mRNA and protein levels after AZD5153 combined with GEM. The further GO, KEGG and GSEA analysis, together with western blotting found that the IL6-JAK2-STAT3 signaling pathway were down-regulated after combination treatment. Treatment with IL-6 increased p-STAT3, which decreased by combination treatment (Figure S6-7, SExcel 1). Overexpression of MUC2 by drugs or Knockdown of MUC2 using siRNA also reverse the expression of p-STAT3 caused by the combination treatment (Figure S8). The MUC2 protein directly mediates Chk2/STAT3/CREB/ATF-1 signaling and E-cadherin expression in colon cancer. Based on STRING database website, physical interactions between MUC2, TP53, CREB1, CDH1, STAT3, IL-6, and CHEK2 was found. MUC2 plays an important role in IL-6 signaling during colon cancer metastasis [47]. However, what role does MUC2 play in our study, and whether the JAK2-STAT3 signaling pathway plays a role with MUC2 is still unknown. More studies should be conducted in the future to explore it. Taken together, these data support the development of a clinical trial combining AZD5153 and gemcitabine for pancreatic cancer treatment.

## Conclusions

In this study, we demonstrated that AZD5153 showed the synergistic effects in combined with GEM in vitro and in vivo. Further study indicated that ERK/mTOR signaling pathway involved in it. It is worth noting that the AZD5153 target gene BRD4 was unchanged, while the MUC2 expression is remarkably down-regulated after combination treatment. Our research suggested that AZD5153 may be a predictive GEM-sensitive drug in pancreatic cancer, and MUC2 may be serving as a key target in this progress.

## Supplementary Information

Supplementary Material 1

## Data Availability

No datasets were generated or analysed during the current study.

## References

[1] Vincent A, Herman J, Schulick R, Hruban RH, Goggins M. Pancreatic cancer. Lancet. 2011;378:607–20. 21620466 10.1016/S0140-6736(10)62307-0 PMC3062508

[2] Zhang B, Xiao J, Cheng X, Liu T. MAL2 interacts with IQGAP1 to promote pancreatic cancer progression by increasing ERK1/2 phosphorylation. Biochem Biophys Res Commun. 2021;554:63–70. 33780861 10.1016/j.bbrc.2021.02.146

[3] Zhu H, Wei M, Xu J, Hua J, Liang C, Meng Q, Zhang Y, Liu J, Zhang B, Yu X, Shi S. PARP inhibitors in pancreatic cancer: molecular mechanisms and clinical applications. Mol Cancer. 2020;19:49. 32122376 10.1186/s12943-020-01167-9 PMC7053129

[4] Lin QJ, Yang F, Jin C, Fu DL. Current status and progress of pancreatic cancer in China. World J Gastroenterol. 2015;21:7988–8003. 26185370 10.3748/wjg.v21.i26.7988 PMC4499341

[5] Zeng S, Pottler M, Lan B, Grutzmann R, Pilarsky C, Yang H. Chemoresistance in pancreatic cancer. Int J Mol Sci. 2019;20:4504. 31514451 10.3390/ijms20184504 PMC6770382

[6] Sarvepalli D, Rashid MU, Rahman AU, Ullah W, Hussain I, Hasan B, Jehanzeb S, Khan AK, Jain AG, Khetpal N, Ahmad S. Gemcitabine: a review of chemoresistance in pancreatic cancer. Crit Rev Oncog. 2019;24:199–212. 31679214 10.1615/CritRevOncog.2019031641

[7] Miller AL, Garcia PL, Yoon KJ. Developing effective combination therapy for pancreatic cancer: an overview. Pharmacol Res. 2020;155: 104740. 32135247 10.1016/j.phrs.2020.104740 PMC7365261

[8] Samanta K, Setua S, Kumari S, Jaggi M, Yallapu MM, Chauhan SC. Gemcitabine combination nano therapies for pancreatic cancer. Pharmaceutics. 2019;11:574. 31689930 10.3390/pharmaceutics11110574 PMC6920852

[9] Rhyasen GW, Hattersley MM, Yao Y, Dulak A, Wang W, Petteruti P, Dale IL, Boiko S, Cheung T, Zhang J, Wen S, Castriotta L, Lawson D, Collins M, Bao L, Ahdesmaki MJ, Walker G, O’Connor G, Yeh TC, Rabow AA, Dry JR, Reimer C, Lyne P, Mills GB, Fawell SE, Waring MJ, Zinda M, Clark E, Chen H. A novel bivalent BET bromodomain inhibitor highly active against hematologic malignancies. Mol Cancer Ther. 2016;AZD5153:2563–74. 10.1158/1535-7163.MCT-16-0141 27573426

[10] Sun Y, Han J, Wang Z, Li X, Sun Y, Hu Z. Safety and efficacy of bromodomain and extra-terminal inhibitors for the treatment of hematological malignancies and solid tumors: a systematic study of clinical trials. Front Pharmacol. 2020;11: 621093. 33574760 10.3389/fphar.2020.621093 PMC7870522

[11] Zhang P, Li R, Xiao H, Liu W, Zeng X, Xie G, Yang W, Shi L, Yin Y, Tao K. BRD4 inhibitor AZD5153 suppresses the proliferation of colorectal cancer cells and sensitizes the anticancer effect of PARP inhibitor. Int J Biol Sci. 2019;15:1942–54. 31523195 10.7150/ijbs.34162 PMC6743290

[12] Shen G, Chen J, Zhou Y, Wang Z, Ma Z, Xu C, Jiang M. AZD5153 inhibits prostate cancer cell growth in vitro and in vivo. Cell Physiol Biochem. 2018;50:798–809. 30308485 10.1159/000494244

[13] Li X, Fu Y, Yang B, Guo E, Wu Y, Huang J, Zhang X, Xiao R, Li K, Wang B, Hu J, Sun C, Chen G. BRD4 inhibition by AZD5153 promotes antitumor immunity via depolarizing M2 macrophages. Front Immunol. 2020;11:89. 32184777 10.3389/fimmu.2020.00089 PMC7058627

[14] Zhang J, Dulak AM, Hattersley MM, Willis BS, Nikkila J, Wang A, Lau A, Reimer C, Zinda M, Fawell SE, Mills GB, Chen H. Brd4 facilitates replication stress-induced DNA damage response. Oncogene. 2018;37:3763–77. 29636547 10.1038/s41388-018-0194-3 PMC6101970

[15] Yin Y, Liu W, Shen Q, Zhang P, Wang L, Tao R, Li H, Ma X, Zeng X, Cheong JH, Song S, Ajani JA, Mills GB, Tao K, Peng G. The DNA endonuclease Mus81 regulates ZEB1 expression and serves as a target of BET4 inhibitors in gastric cancer. Mol Cancer Ther. 2019;18:1439–50. 31142662 10.1158/1535-7163.MCT-18-0833 PMC8345820

[16] Huang M, Zeki J, Sumarsono N, Coles GL, Taylor JS, Danzer E, Bruzoni M, Hazard FK, Lacayo NJ, Sakamoto KM, Dunn JCY, Spunt SL, Chiu B. Epigenetic targeting of TERT-associated gene expression signature in human neuroblastoma with TERT overexpression. Cancer Res. 2020;80:1024–35. 31900258 10.1158/0008-5472.CAN-19-2560 PMC7056551

[17] Tan B, Huang Y, Lan L, Zhang B, Ye L, Yan W, Wang F, Lin N. Bruceine D induces apoptosis in human non-small cell lung cancer cells through regulating JNK pathway. Biomed Pharmacother. 2019;117: 109089. 31226632 10.1016/j.biopha.2019.109089

[18] Zhang D, Zhang B, Zhou LX, Zhao J, Yan YY, Li YL, Zeng JM, Wang LL, Yang B, Lin NM. Deacetylisovaltratum disrupts microtubule dynamics and causes G2/M-phase arrest in human gastric cancer cells in vitro. Acta Pharmacol Sin. 2016;37:1597–605. 27665846 10.1038/aps.2016.91 PMC5260834

[19] Ye Z, Zhuo Q, Hu Q, Xu X, Mengqi L, Zhang Z, Xu W, Liu W, Fan G, Qin Y, Yu X, Ji S. FBW7-NRA41-SCD1 axis synchronously regulates apoptosis and ferroptosis in pancreatic cancer cells. Redox Biol. 2021;38:101807. 33271455 10.1016/j.redox.2020.101807 PMC7710650

[20] Nicolle R, Gayet O, Duconseil P, Vanbrugghe C, Roques J, Bigonnet M, Blum Y, Elarouci N, Armenoult L, Ayadi M, de Reynies A, Puleo F, Augustin J, Emile JF, Svrcek M, Arsenijevic T, Hammel P, Giovannini M, Grandval P, Dahan L, Moutardier V, Gilabert M, Van Laethem JL, Bachet JB, Cros J, Iovanna J, Dusetti NJ. A transcriptomic signature to predict adjuvant gemcitabine sensitivity in pancreatic adenocarcinoma. Ann Oncol. 2021;32(2):250–60. 33188873 10.1016/j.annonc.2020.10.601

[21] Cazes A, Betancourt O, Esparza E, Mose ES, Jaquish D, Wong E, Wascher AA, Tiriac H, Gymnopoulos M, Lowy AM. A MET targeting antibody-drug conjugate overcomes gemcitabine resistance in pancreatic cancer. Clin Cancer Res. 2021;27:2100–10. 33451980 10.1158/1078-0432.CCR-20-3210

[22] Ryu WJ, Han G, Lee SH, Choi KY. Suppression of Wnt/beta-catenin and RAS/ERK pathways provides a therapeutic strategy for gemcitabine-resistant pancreatic cancer. Biochem Biophys Res Commun. 2021;549:40–6. 33662667 10.1016/j.bbrc.2021.02.076

[23] Wolfe AR, Robb R, Hegazi A, Abushahin L, Yang L, Shyu DL, Trevino JG, Cruz-Monserrate Z, Jacob JR, Palanichamy K, Chakravarti A, Williams TM. Altered gemcitabine and Nab-paclitaxel scheduling improves therapeutic efficacy compared with standard concurrent treatment in preclinical models of pancreatic cancer. Clin Cancer Res. 2021;27:554–65. 33087331 10.1158/1078-0432.CCR-20-1422 PMC7855515

[24] Matsumoto R, Hamada S, Tanaka Y, Taguchi K, Yamamoto M, Masamune A. Nrf2 Depletion Sensitizes Pancreatic Cancer Cells to Gemcitabine via Aldehyde Dehydrogenase 3a1 Repression, J Pharmacol Exp Ther (2021) JPET-AR-2021-000744. 10.1124/jpet.121.000744 34321315

[25] Waissi W, Nicol A, Jung M, Rousseau M, Jarnet D, Noel G, Burckel H. Radiosensitizing pancreatic cancer with PARP inhibitor and gemcitabine: an in vivo and a whole-transcriptome analysis after proton or photon irradiation. Cancers (Basel). 2021;13: 13:527. 33573176 10.3390/cancers13030527 PMC7866541

[26] Wang S, Feng W, Wang W, Ye X, Chen H, Yu C. Girdin knockdown increases gemcitabine chemosensitivity to pancreatic cancer by modulating autophagy. Front Oncol. 2021;11: 618764. 33854963 10.3389/fonc.2021.618764 PMC8039524

[27] Waissi W, Ame JC, Mura C, Noel G, Burckel H. Gemcitabine-based chemoradiotherapy enhanced by a PARP inhibitor in pancreatic cancer cell lines. Int J Mol Sci. 2021;22: 6825. 34201963 10.3390/ijms22136825 PMC8269291

[28] Li Y, Xi Z, Chen X, Cai S, Liang C, Wang Z, Li Y, Tan H, Lao Y, Xu H. Natural compound Oblongifolin C confers gemcitabine resistance in pancreatic cancer by downregulating src/mapk/erk pathways. Cell Death Dis. 2018;9:538. 29749405 10.1038/s41419-018-0574-1 PMC5970202

[29] Wang W, Qin JJ, Voruganti S, Nijampatnam B, Velu SE, Ruan KH, Hu M, Zhou J, Zhang R. Discovery and characterization of dual inhibitors of MDM2 and NFAT1 for pancreatic cancer therapy. Cancer Res. 2018;78:5656–67. 30217928 10.1158/0008-5472.CAN-17-3939 PMC6435280

[30] Zhou Q, Tu X, Hou X, Yu J, Zhao F, Huang J, Kloeber J, Olson A, Gao M, Luo K, Zhu S, Wu Z, Zhang Y, Sun C, Zeng X, Schoolmeester KJ, Weroha JS, Hu X, Jiang Y, Wang L, Mutter RW. Syk-dependent homologous recombination activation promotes cancer resistance to DNA targeted therapy. Drug Resist Updat. 2024;74: 101085. 38636338 10.1016/j.drup.2024.101085 PMC11095636

[31] Jayaprakash Mandal TN, Jones JM, Liberto S, Gaillard T-L, Wang I-M, Shih. Dual Inhibition of SYK and EGFR overcomes chemoresistance by inhibiting CDC6 and blocking DNA replication. Cancer Res 2024 Aug 9. 10.1158/0008-5472.CAN-24-0769. Online ahead of print. 10.1158/0008-5472.CAN-24-0769 39120597

[32] Jan A, Burger A, Wiestner. Targeting B cell receptor signalling in cancer: preclinical and clinical advances. Nat Rev Cancer. 2018;18(3):148–67. 29348577 10.1038/nrc.2017.121

[33] Zhou XY, Ma EH, Zhang YY, Xing YJ, Xu WB, Zhou LCH 1, Zhang XR, Jiang CR, Xu K, Wang H, Zheng SH. NIR-Actuated targeted Janus nanomotors remodel immunosuppressive tumor microenvironment for augmented cancer immunotherapy. Adv Healthc Mater. 2024;13(2):e2302272. 10.1002/adhm.202302272 37824087

[34] Aubrey L, Miller PL, Garcia SC, Fehling TL, Gamblin, Rebecca B, Vance LN, Council D, Chen, Eddy S, Yang. Robert C A M Van waardenburg, Karina J yoon. The BET inhibitor JQ1 augments the antitumor efficacy of gemcitabine in preclinical models of pancreatic cancer. Cancers (Basel). 2021;13(14):3470. 34298684 10.3390/cancers13143470 PMC8303731

[35] Wang J, Xu YZ, Rao XR, Zhang RG, Tang J, Zhang D, Jie XH, Zhu KK, Wang X, Xu YH, Zhang S, Dong XR, Zhang T, Yang KY, Xu SB, Meng R, Wu G. BRD4-IRF1 axis regulates chemoradiotherapy-induced PD-L1 expression and immune evasion in non-small cell lung cancer. Clin Transl Med. 2022;12(1): e718. 35083874 10.1002/ctm2.718 PMC8792480

[36] P Erika, Js Hamilton, Am Wang, Mr Oza, Sv Patel, T Ulahannan, Jl Bauer, M Karlix, Gillian M Hattersley, P Littlewood, Jamal Saeh Mitchell, Pouliot Gayle P, Moore Kathleen N. First-in-human study of AZD5153, a small-molecule inhibitor of bromodomain protein 4, in patients with relapsed/refractory malignant solid tumors and lymphoma. Mol Cancer Ther. 2023;22(10):1154–65. 37486983 10.1158/1535-7163.MCT-23-0065 PMC10544002

[37] Massihnia D, Avan A, Funel N, Maftouh M, van Krieken A, Granchi C, Raktoe R, Boggi U, Aicher B, Minutolo F, Russo A, Leon LG, Peters GJ, Giovannetti E. Phospho-Akt overexpression is prognostic and can be used to tailor the synergistic interaction of Akt inhibitors with gemcitabine in pancreatic cancer. J Hematol Oncol. 2017;10:9. 28061880 10.1186/s13045-016-0371-1 PMC5219723

[38] Wang Z, Luo G, Qiu Z. Akt inhibitor MK-2206 reduces pancreatic cancer cell viability and increases the efficacy of gemcitabine. Oncol Lett. 2020;19:1999–2004. 32194695 10.3892/ol.2020.11300 PMC7039141

[39] Hirai H, Sootome H, Nakatsuru Y, Miyama K, Taguchi S, Tsujioka K, Ueno Y, Hatch H, Majumder PK, Pan BS, Kotani H. MK-2206, an allosteric Akt inhibitor, enhances antitumor efficacy by standard chemotherapeutic agents or molecular targeted drugs in vitro and in vivo. Mol Cancer Ther. 2010;9:1956–67. 20571069 10.1158/1535-7163.MCT-09-1012

[40] Wei R, Penso NEC, Hackman RM, Wang Y, Mackenzie GG. Epigallocatechin-3-gallate (EGCG) suppresses pancreatic cancer cell growth, invasion, and migration partly through the inhibition of Akt pathway and epithelial-mesenchymal transition: enhanced efficacy when combined with gemcitabine. Nutrients. 2019;11:1856. 31405071 10.3390/nu11081856 PMC6722696

[41] Velcich A, Yang WC, Heyer J, Fragale A, Nicholas C, Viani S, Kucherlapati R, Lipkin M, Yang K. Leonard augenlicht, colorectal cancer in mice genetically deficient in the mucin Muc2. Science. 2002;295(5560):1726–9. 11872843 10.1126/science.1069094

[42] Dai F, Dong S, Rong Z, Xuan Q, Chen P, Chen M, Fan Y, Gao Q. Expression of inositol-requiring enzyme 1β is downregulated in azoxymethane/dextran sulfate sodium-induced mouse colonic tumors. Exp Ther Med. 2019;17(4):3181–8. 30936991 10.3892/etm.2019.7317 PMC6434252

[43] Cardillo MR, Castagna G, Memeo L, De Bernardinis E, Di Silverio F. Epidermal growth factor receptor, MUC-1 and MUC-2 in bladder cancer. J Exp Clin Cancer Res. 2000;19(2):225–33. 10965823

[44] Anuk T, Beseren H, Polat Y, Ozden O, Kalayci T. Clinicopathological factors affecting mucin 1, mucin 2, and mucin 5AC staining in patients who underwent resection for colorectal cancer. J Coll Physicians Surg Pak. 2023;33(3):335–40. 36945166 10.29271/jcpsp.2023.03.335

[45] Zhou HY, Zhu YW, Wei FF, Shao YT, Pan JJ, Wang G, Xu KQ, Cheng YQ. Significance of MUC2 gene methylation detection in pancreatic cancer diagnosis. Pancreatology. 2019;19(8):1049–53. 31590960 10.1016/j.pan.2019.09.012

[46] Yokoyama S, Hamada T, Higashi M, Matsuo K, Maemura K, Kurahara H, Horinouchi M, Hiraki T, Sugimoto T, Akahane T, Yonezawa S, Kornmann M, Batra SK, Hollingsworth MA, Tanimoto A. Predicted prognosis of patients with pancreatic cancer by machine learning. Clin Cancer Res. 2020;26(10):2411–21. 31992588 10.1158/1078-0432.CCR-19-1247

[47] Hsu HP, Lai MD, Lee JC, Yen MC, Weng TY, Chen WC, Fang JH, Chen YL. Mucin 2 silencing promotes colon cancer metastasis through interleukin-6 signaling. Sci Rep. 2017;7(1):5823. 28725043 10.1038/s41598-017-04952-7 PMC5517441

