# Supplementary figures and images for: AZD5153 enhances the chemo-sensitivity of gemcitabine on pancreatic cancer cells in vitro and in vivo
Source: Cancer Cell Int. 2025 Aug 26;25:315. doi: 10.1186/s12935-025-03952-2 (PMC12379372; doi:10.1186/s12935-025-03952-2)

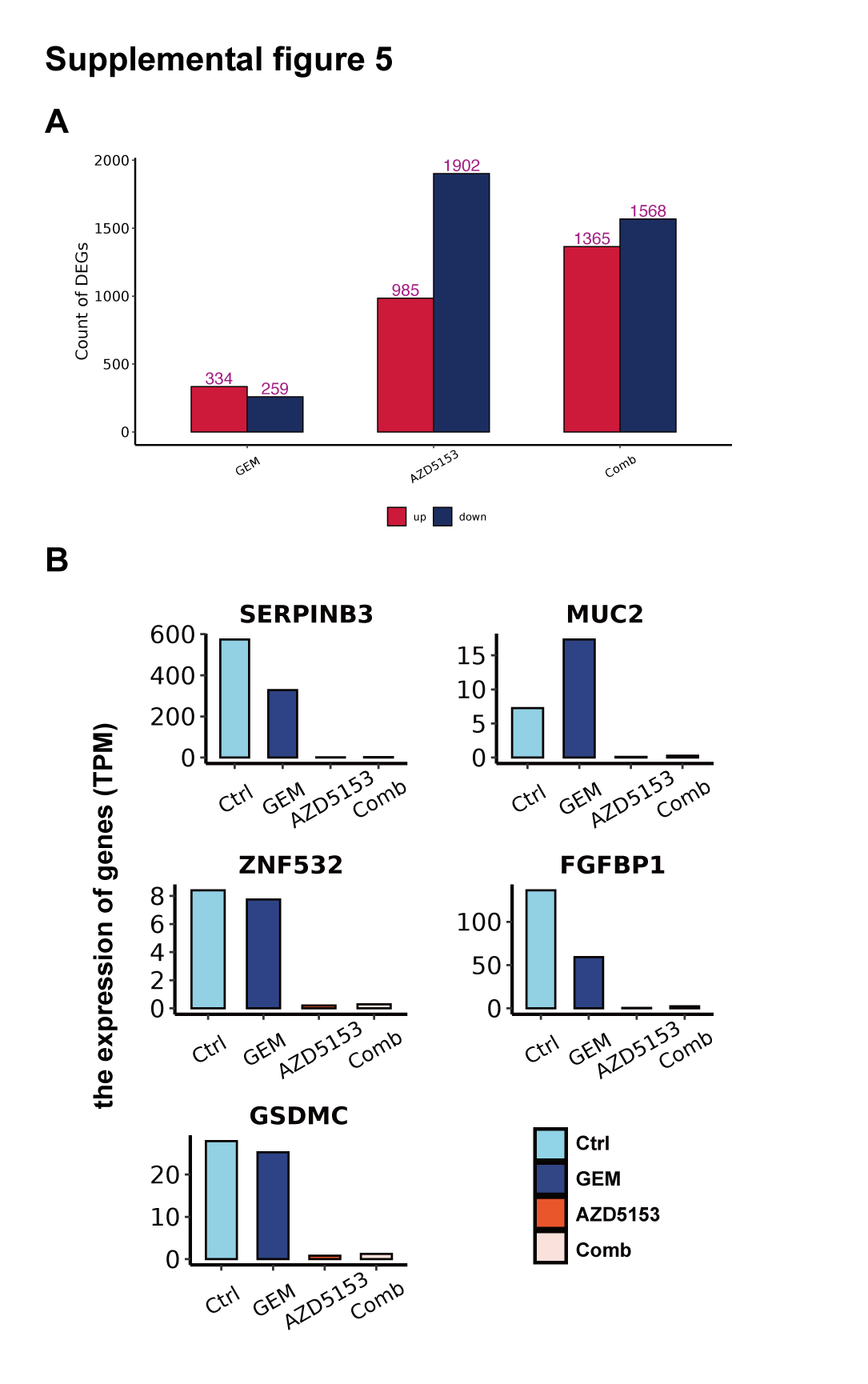

Supplement: Supplementary file 1 — Supplementary Material 1 [file 12935_2025_3952_MOESM1_ESM.zip › 20250718 Stable+Sfigures/SFig 5.tif]

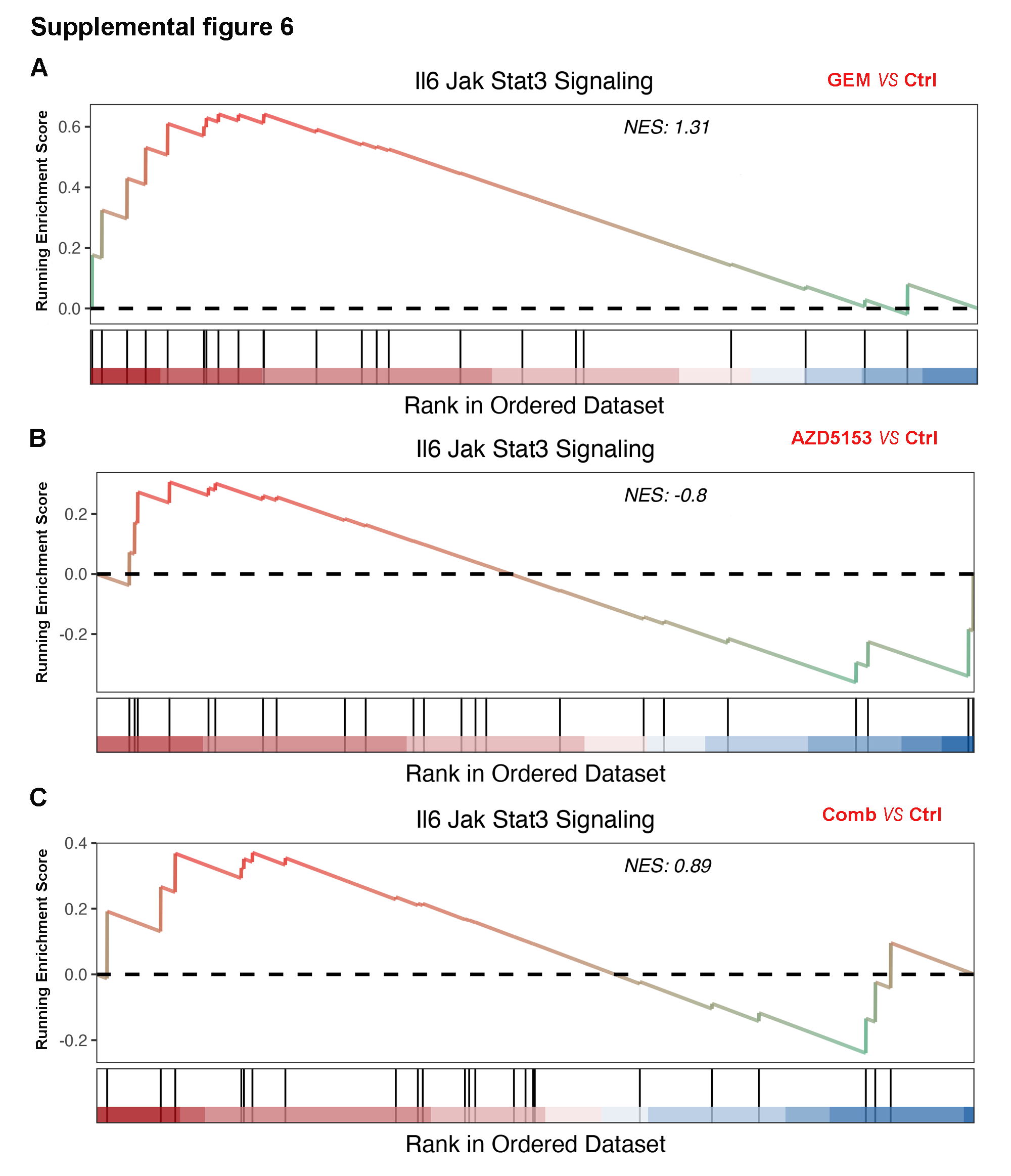

Supplement: Supplementary file 1 — Supplementary Material 1 [file 12935_2025_3952_MOESM1_ESM.zip › 20250718 Stable+Sfigures/SFig 6.tif]

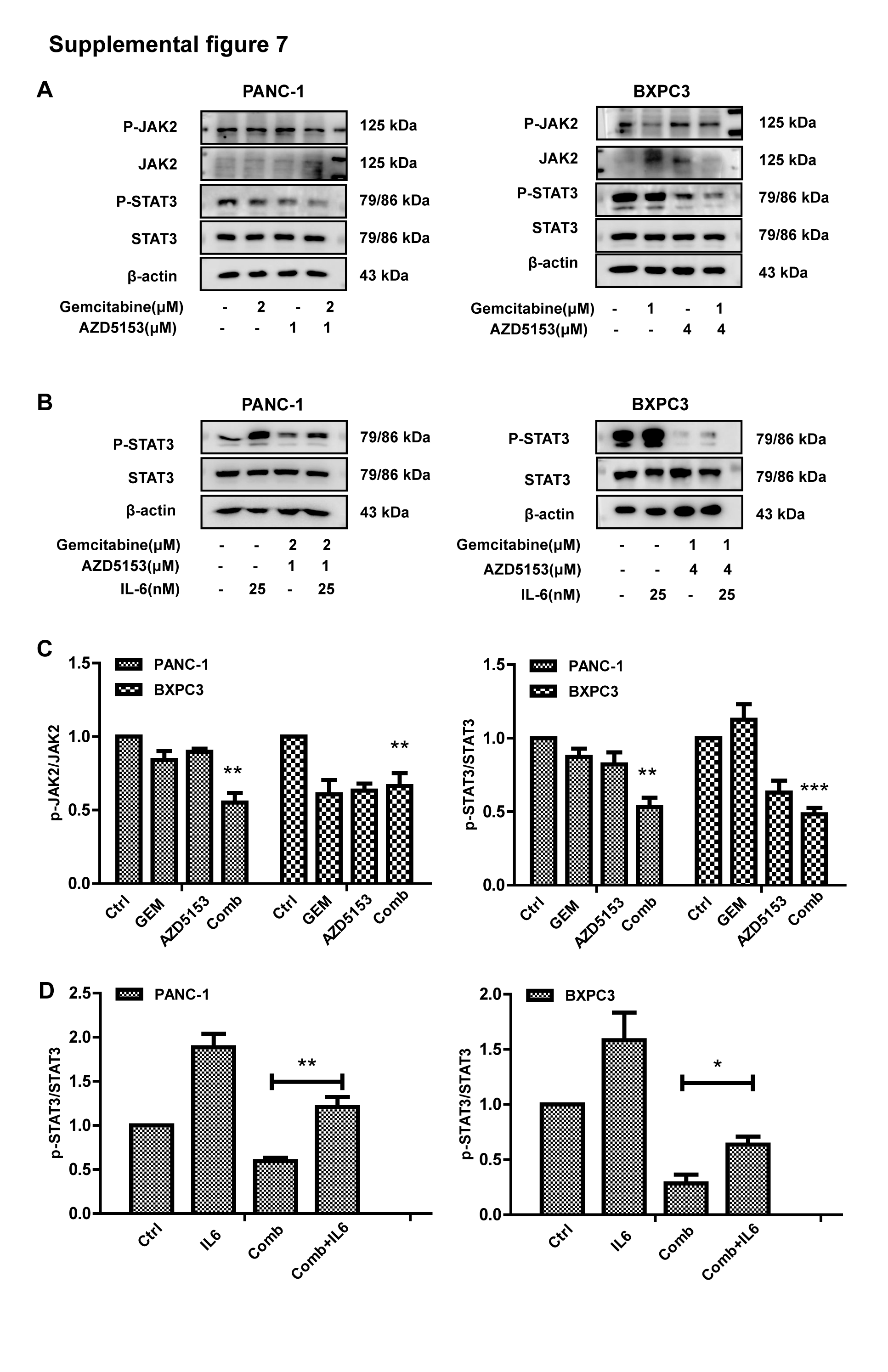

Supplement: Supplementary file 1 — Supplementary Material 1 [file 12935_2025_3952_MOESM1_ESM.zip › 20250718 Stable+Sfigures/SFig 7.tif]

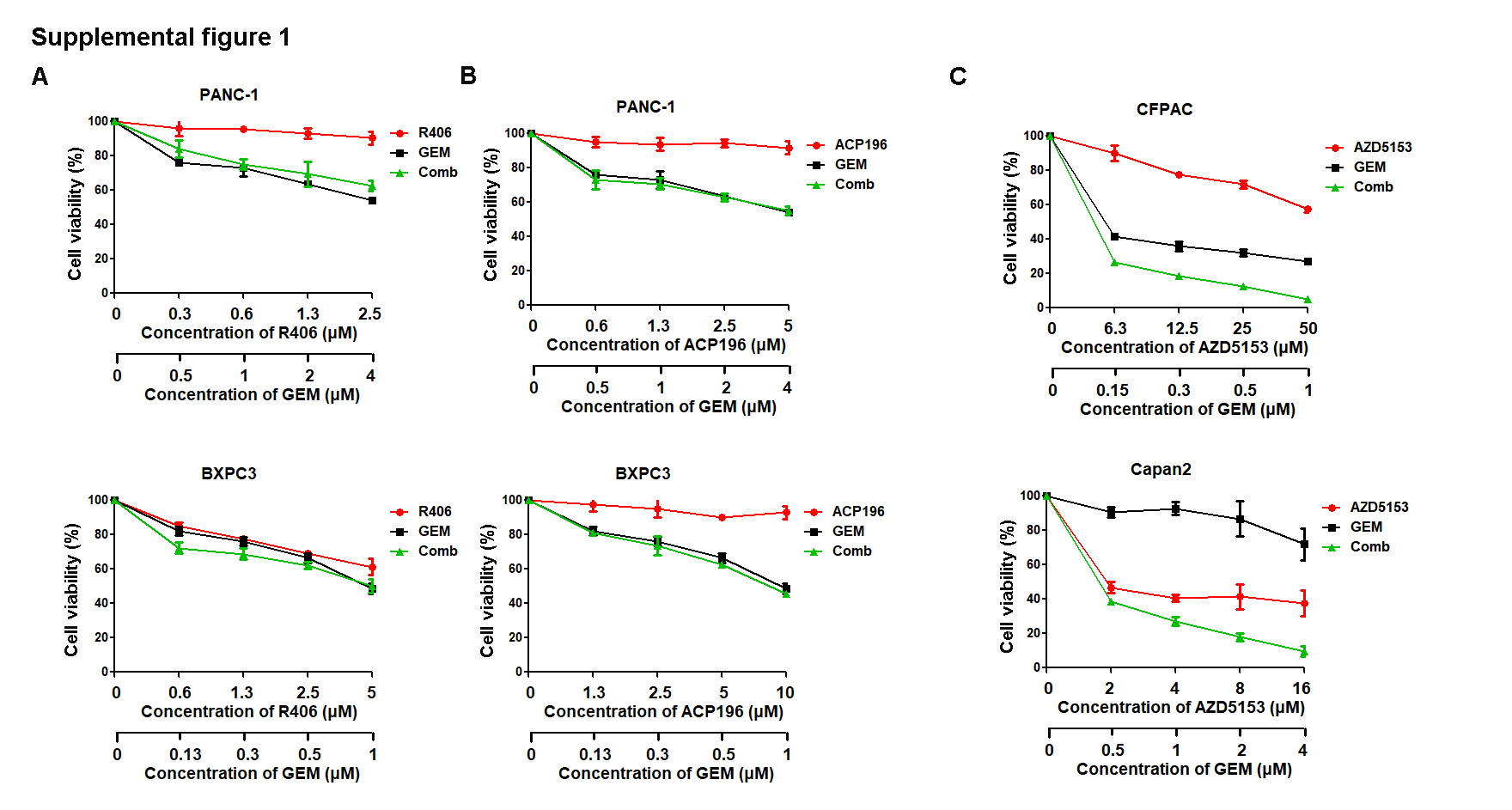

Supplement: Supplementary file 1 — Supplementary Material 1 [file 12935_2025_3952_MOESM1_ESM.zip › 20250718 Stable+Sfigures/Sfig 1.tif]

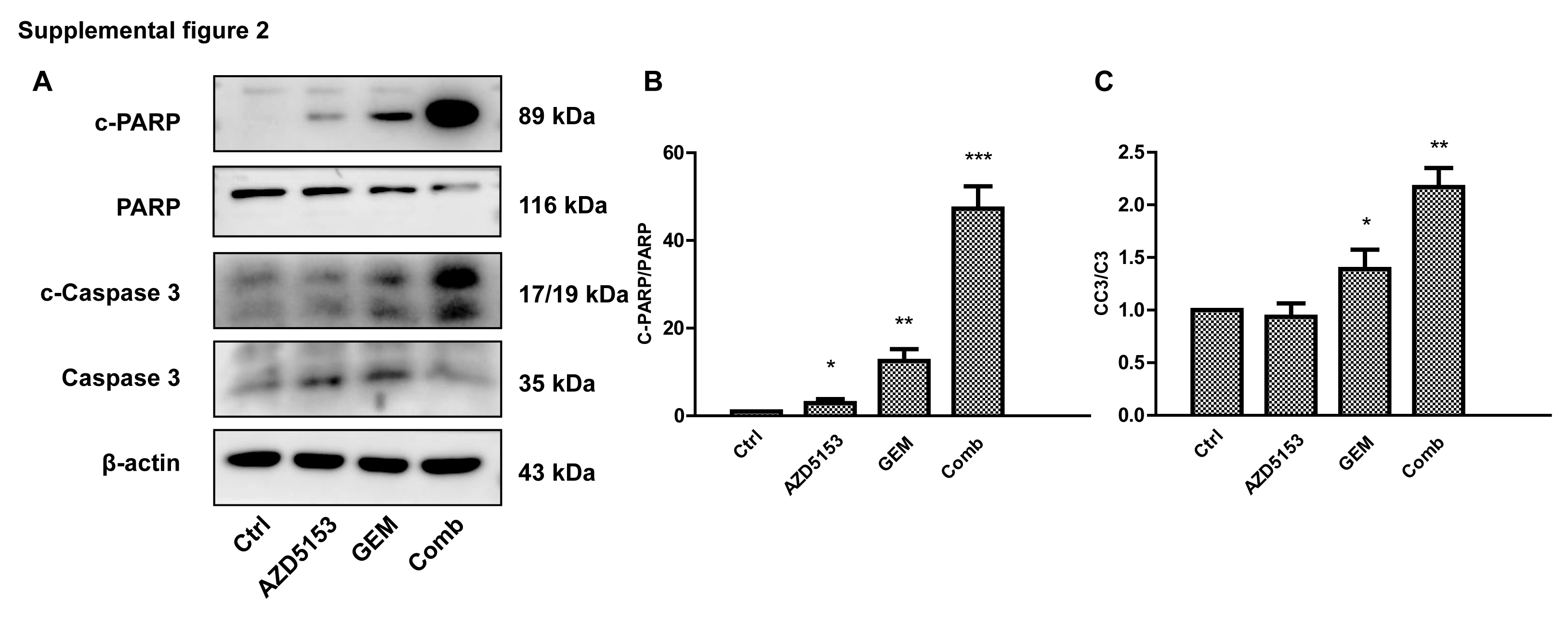

Supplement: Supplementary file 1 — Supplementary Material 1 [file 12935_2025_3952_MOESM1_ESM.zip › 20250718 Stable+Sfigures/Sfig 2.tif]

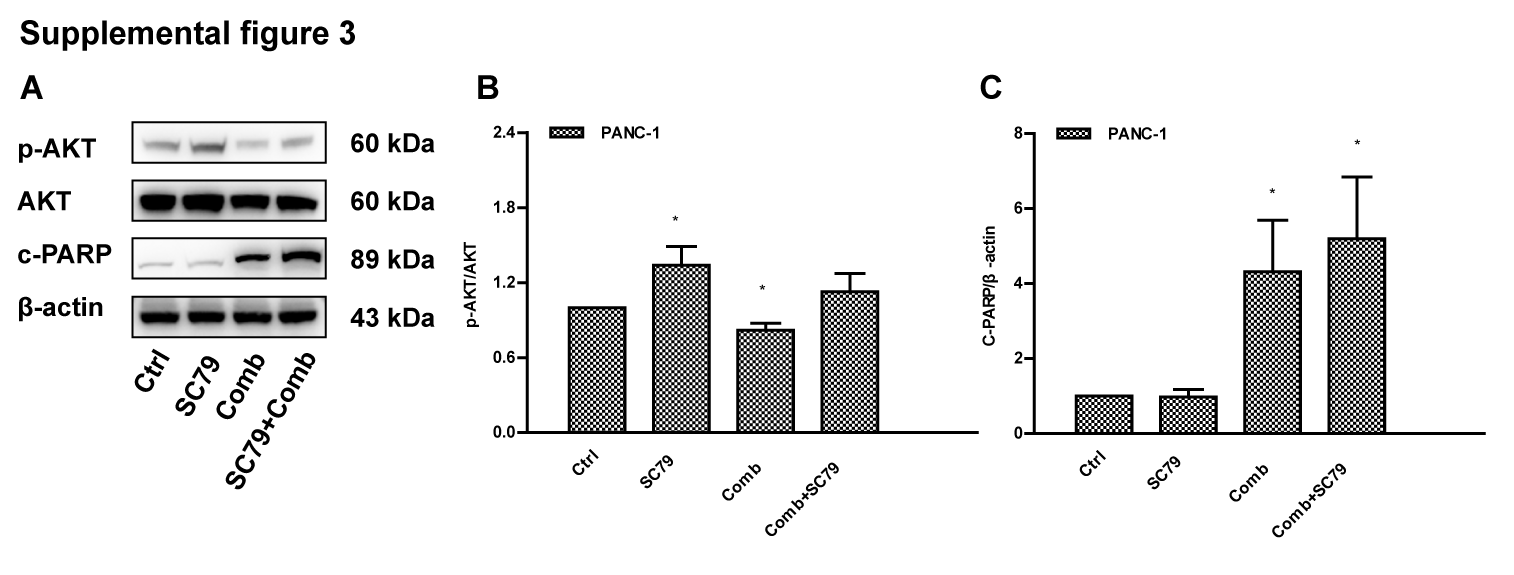

Supplement: Supplementary file 1 — Supplementary Material 1 [file 12935_2025_3952_MOESM1_ESM.zip › 20250718 Stable+Sfigures/Sfig 3.tif]

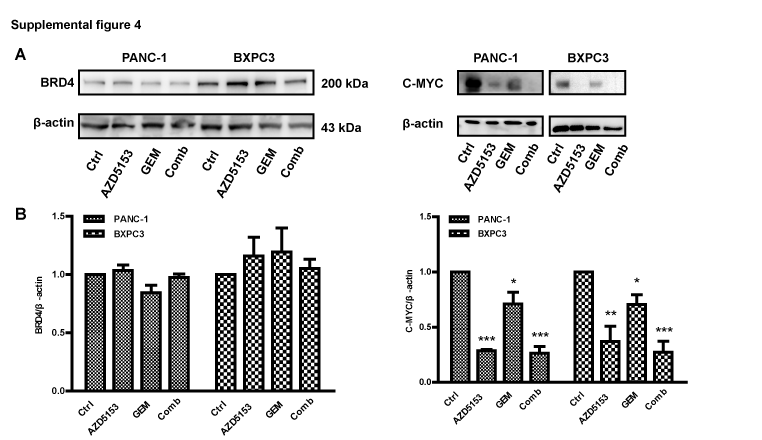

Supplement: Supplementary file 1 — Supplementary Material 1 [file 12935_2025_3952_MOESM1_ESM.zip › 20250718 Stable+Sfigures/Sfig 4.tif]

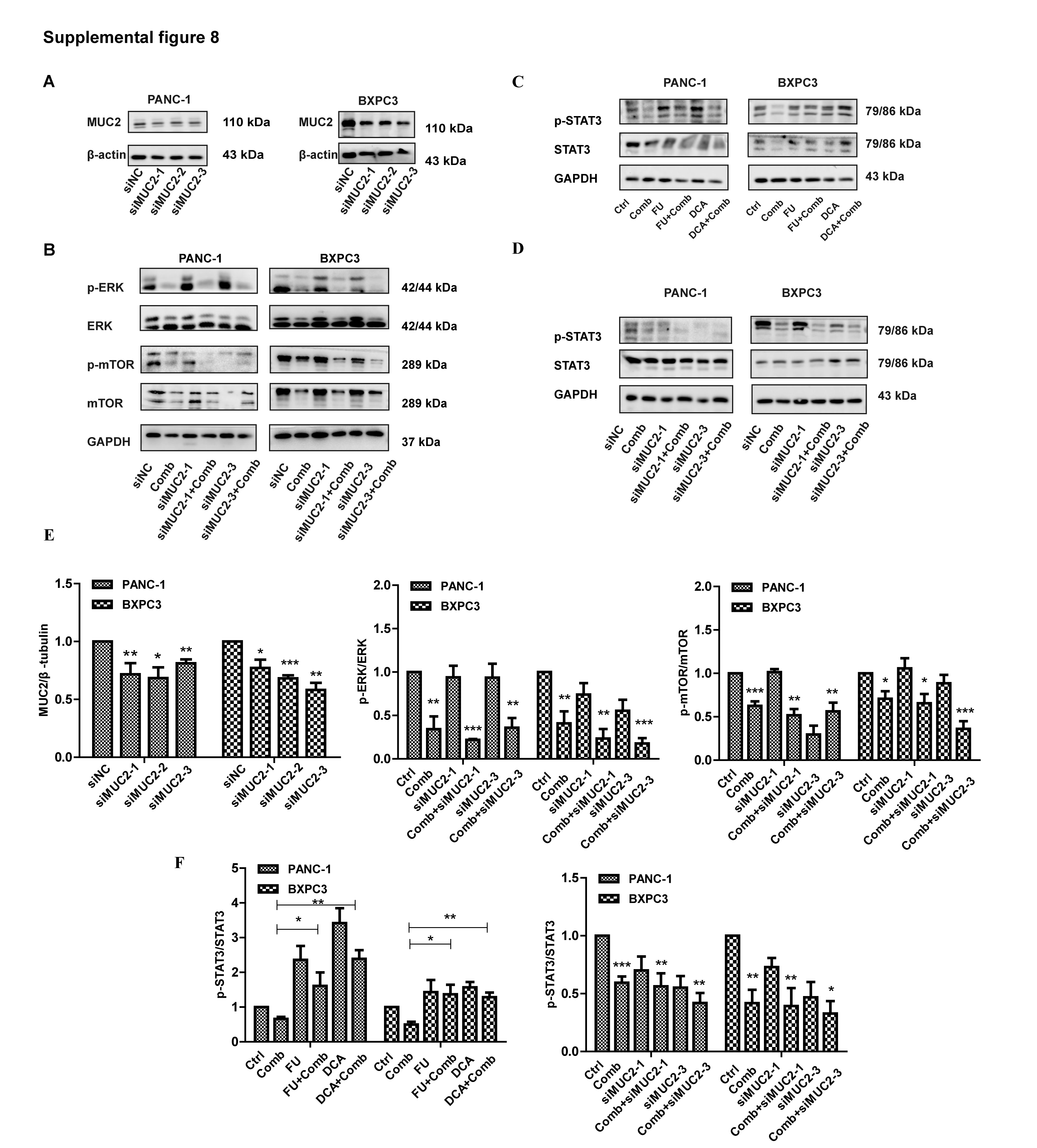

Supplement: Supplementary file 1 — Supplementary Material 1 [file 12935_2025_3952_MOESM1_ESM.zip › 20250718 Stable+Sfigures/Sfig 8.tif]
