# Supplementary material for: AZD5153 enhances the chemo-sensitivity of gemcitabine on pancreatic cancer cells in vitro and in vivo
Source: Cancer Cell Int. 2025 Aug 26;25:315. doi: 10.1186/s12935-025-03952-2 (PMC12379372; doi:10.1186/s12935-025-03952-2)
Supplement: Supplementary file 1 — Supplementary Material 1 [file 12935_2025_3952_MOESM1_ESM.zip › 20250718 Stable+Sfigures/Stable1-1+Sfigures-clean version.doc]

**Supplemental Table 1**

CI values of AZD5153 at concentrations applied in combination with gemcitabine in BXPC3 and PANC-1 cells

| **BXPC3** | | | **PANC-1** | | |
| --- | --- | --- | --- | --- | --- |
| **AZD5153 (μM)** | **gem (μM)** | **CI** | **AZD5153 (μM)** | **gem (μM)** | **CI** |
| 0.5 | 0.125 | 0.42 | 0.25 | 0.5 | 0.23 |
| 1 | 0.25 | 0.32 | 0.5 | 1 | 0.22 |
| 2 | 0.5 | 0.24 | 1 | 2 | 0.32 |
| 4 | 1 | 0.31 | 2 | 4 | 0.48 |

A CI less than 0.90 indicates synergism; 0.10, very strong synergism; 0.10-0.30, strong synergism; 0.30-0.70, synergism; 0.70-0.85, moderate synergism; 0.85-0.9, slight synergism; 0.90-1.10, additive; and more than 1.10, antagonism.

**Supplemental Figure 1.** **R406/ACP196/AZD5153, GEM, and their combination inhibited cell proliferation *in vitro*.** Cells were treated with PBS, R406/ACP196/AZD5153, GEM, or their combination for 48 h, and then the absorbance was measured.

**Supplemental Figure 2. The expression of apoptosis-related proteins in tumor tissues.** Levels of PARP, c-PARP, Caspase 3 and c-Caspase 3 in different groups were measured using western blotting. β-actin is the loading control. The results are presented as the means ± SD of 3 independent experiments.

**Supplemental Figure 3. SC79 could not reverse the expression of c-PARP induced by AZD5153 and GEM**. Cells were pretreated with SC79, then treated with AZD5153 and GEM for 24 h. Western blotting analysis was performed to detect the expression of AKT, p-AKT (S473), c-PARP, and β-actin, the loading control. The results are presented as the means ± SD of 3 independent experiments.

**Supplemental Figure 4.** **AZD5153 combined with GEM did not change the expression of BRD4.** (A) Cells were treated with AZD5153, GEM, or their combination for 48h. Western blotting analysis was performed to detect the levels of BRD4, C-MYC and β-actin was used asthe loading control. (B) The ratios of BRD4/β-actin, C-MYC/β-actin were quantified by densitometry based on immunoblot images. The results are presented as the means ± SD of 3 independent experiments. Significance was determined using Student's t-test (**p*<0.05, ***p*<0.01, ****p*<0.001 *vs.* Ctrl group.

**Supplemental Figure 5.** **Differential gene expression in pancreatic cancer cells.** RNA-sequencing was conducted to detect the expression of gene expression. (A) The all changed genes were showed in different groups. (B) The expression of five potential genes in four groups.

**Supplemental Figure 6.** **IL6-JAK-STAT3 signaling pathway were analyzed by GSEA**. (A) GEM *vs* Ctrl. (B) AZD5153 *vs* Ctrl. (C) Comb *vs* Ctrl.

**Supplemental Figure 7. The expression of JAK2, STAT3 in pancreatic cancer cells.** (A) Cells were treated with AZD5153, GEM, or their combination for 4 h. Western blotting analysis was performed to detect the levels of JAK2, p-JAK2, STAT3, p-STAT3, and β-actin, the loading control. (B) Cells were pretreated with IL6 (25 nM) and then treated with AZD5153 and GEM for 4 h. Western blotting analysis was performed to detect the expression of STAT3, p-STAT3, and β-actin was used as a loading control. (C-D) The ratios of p-JAK2/JAK2, p-STAT3/STAT3 were quantified by densitometry based on immunoblot images. The results are presented as the means±SD of 3 independent experiments. Significance was determined by One-way ANOVA analysis (**p*<0.05, ***p*<0.01, ****p*<0.001 *vs.* Ctrl group in C or *vs.* Comb group in D).

**Supplemental Figure 8. The expression of pERK/mTOR and JAK2/STAT3 signaling pathway in pancreatic cancer cells.** (A-B, D) Western blotting analysis was performed to detect the levels of MUC2, p-ERK, ERK, p-mTOR, mTOR after knockdown of MUC2 in PANC-1 and BXPC3 cells. (C) Western blotting analysis was performed to detect the levels of p-STAT3, STAT3 after knockdown of MUC2 or FU/DCA treatment in PANC-1 and BXPC3 cells. β-actin or GAPDH was used as a loading control. (E-F) The ratios of p-ERK/ERK, p-mTOR/mTOR were quantified by densitometry based on immunoblot images. The results are presented as the means±SD of 3 independent experiments. Significance was determined by One-way ANOVA analysis (**p*<0.05, ***p*<0.01, ****p*<0.001 *vs.* Ctrl/NC group in E, F right or *vs.* Comb group in F left).
